# Microbiota and Metabolite Profiling as Markers of Mood Disorders: A Cross-Sectional Study in Obese Patients

**DOI:** 10.3390/nu14010147

**Published:** 2021-12-29

**Authors:** Quentin Leyrolle, Renata Cserjesi, Romane Demeure, Audrey M. Neyrinck, Camille Amadieu, Julie Rodriguez, Olli Kärkkäinen, Kati Hanhineva, Nicolas Paquot, Miriam Cnop, Patrice D. Cani, Jean-Paul Thissen, Laure B. Bindels, Olivier Klein, Olivier Luminet, Nathalie M. Delzenne

**Affiliations:** 1 Metabolism and Nutrition Research Group, Louvain Drug Research Institute, UCLouvain, 1200 Brussels, Belgium; quentin.leyrolle@uclouvain.be (Q.L.); romane.demeure@student.uclouvain.be (R.D.); audrey.neyrinck@uclouvain.be (A.M.N.); camille.amadieu@uclouvain.be (C.A.); j.rodriguez@uclouvain.be (J.R.); patrice.cani@uclouvain.be (P.D.C.); laure.bindels@uclouvain.be (L.B.B.); 2 Center for Social and Cultural Psychology, Université Libre de Bruxelles, 1000 Brussels, Belgium; cserjesi.renata@ppk.elte.hu (R.C.); oklein@ulb.ac.be (O.K.); 3 Institute of Psychology, Eötvös Loránd University, 1053 Budapest, Hungary; 4 School of Pharmacy, University of Eastern Finland, 70211 Kuopio, Finland; olli.karkkainen@uef.fi; 5 Food Chemistry and Food Development Unit, Department of Life Technologies, University of Turku, 20014 Turku, Finland; kati.hanhineva@utu.fi; 6 Institute of Public Health and Clinical Nutrition, University of Eastern Finland, 70211 Kuopio, Finland; 7 Laboratory of Immunometabolism and Nutrition, GIGA-Inflammation, Infection & Immunity, University of Liège, 4000 Liège, Belgium; Nicolas.Paquot@ulg.ac.be; 8 ULB Center for Diabetes Research, Medical Faculty, Université Libre de Bruxelles (ULB), 1070 Brussels, Belgium; mcnop@ulb.ac.be; 9 Division of Endocrinology, Erasmus Hospital, Université Libre de Bruxelles, 1070 Brussels, Belgium; 10 WELBIO-Walloon Excellence in Life Sciences and BIOtechnology, UCLouvain, 1200 Brussels, Belgium; 11 Pole of Endocrinology, Diabetes and Nutrition, Institut de Recherche Expérimentale et Clinique IREC, UCLouvain, 1200 Brussels, Belgium; jeanpaul.thissen@uclouvain.be; 12 Research Institute for Psychological Sciences, UCLouvain, 1348 Louvain-la-Neuve, Belgium; olivier.luminet@uclouvain.be

**Keywords:** mood disorders, microbiota, obesity, metabolomics, behaviour

## Abstract

Obesity is associated with an increased risk of several neurological and psychiatric diseases, but few studies report the contribution of biological features in the occurrence of mood disorders in obese patients. The aim of the study is to evaluate the potential links between serum metabolomics and gut microbiome, and mood disturbances in a cohort of obese patients. Psychological, biological characteristics and nutritional habits were evaluated in 94 obese subjects from the Food4Gut study stratified according to their mood score assessed by the Positive and Negative Affect Schedule (PANAS). The fecal gut microbiota and plasma non-targeted metabolomics were analysed. Obese subjects with increased negative mood display elevated levels of *Coprococcus* as well as decreased levels of *Sutterella* and *Lactobacillus*. Serum metabolite profile analysis reveals in these subjects altered levels of several amino acid-derived metabolites, such as an increased level of L-histidine and a decreased in phenylacetylglutamine, linked to altered gut microbiota composition and function rather than to differences in dietary amino acid intake. Regarding clinical profile, we did not observe any differences between both groups. Our results reveal new microbiota-derived metabolites that characterize the alterations of mood in obese subjects, thereby allowing to propose new targets to tackle mood disturbances in this context. Food4gut, clinicaltrial.gov: NCT03852069.

## 1. Introduction

Obesity affects a growing proportion of the world population [1]. This disease is associated with several comorbidities including cancer, diabetes, cardiovascular diseases and neurological disturbances [2]. Obese patients have a marked increased risk in developing depression, anxiety disorders or neurodegenerative diseases [3]. Several studies report that low-grade inflammation occurring in obesity can be responsible for the increased risk of psychiatric disease [4]. The gut microbial dysbiosis, meaning disturbances of the gut microbiota composition and function, has been highlighted in the context of psychiatric disease on the one hand, and in obesity on the other hand [5,6]. Indeed, it has already been shown that the transfer to mice of the gut microbiota from patients presenting a dysbiosis associated to depression and/or psychiatric disorders (such as major depression or alcohol use disorders) recapitulates at least partially the behavioural alterations [7,8]. In obesity, it is now well accepted that the characteristics of the gut microbiota may modulate the production of metabolites that can in turn drive metabolic or inflammatory disturbances [9,10]. The combination of both pathological components probably involves several factors, notably inflammation, hormonal disturbances or insulin-resistance [3]. The gut microbiota is able to produce a wide variety of molecules that can reach the blood and affect the whole-body physiology, including the brain [11]. Among microbiota-derived metabolites, several are thought to exert neuroactive properties including short chain fatty acids (SCFA) or bile acids [12,13]. Recent studies highlighted that some gut-derived metabolites originating from aromatic amino acids metabolism are able to modulate cognition and brain function in obesity [14,15]. Since a crosstalk exists between the gut microbiota and the brain, the circulating metabolome is susceptible to play an important role in psychiatric disorders. To date, little is known regarding the link between the gut microbiome, the blood metabolite composition, and nutrition in obesity-associated mood disturbances. Knowing that diet and microbiome are the strongest determinants of the human serum metabolome [16], the identification of bioactive metabolites issued from the interaction of food components and microbiota could be helpful in proposed biomarkers of psychological health improvement based on nutritional strategies.

In the present paper, we focused on mood disturbances in a cohort of obese patients. We split a population of 94 obese individuals from the Food4Gut cohort based on their mood scores by using the positive and negative affect scale (PANAS). Then we compared the clinical, microbial and metabolite profiles of the patients to highlight potential new targets in the management of psychological disturbances associated with obesity.

## 2. Materials and Methods

### 2.1. Participants

Men and women were recruited in three university hospitals in Belgium (Hôpital Erasme in Brussels, Centre Hospitalier Universitaire in Liège and Cliniques universitaires Saint-Luc Brussels). The original study (Food4Gut cohort) was a 3-month long, multicentric, single-blind, placebo-controlled trial (more details in Supplementary Methods) [17]. Among the 106 patients included in the Food4Gut study, 94 were classified upon their positivity score (see more details in the “psychological measures” section). Gut microbiota composition was available for 86 subjects while metabolomics analysis was conducted only in one of the three centers (the Cliniques universitaires Saint-Luc, Brussels) which correspond to 38 patients (see Supplementary Figure S1 for more details). This study was approved by the “Comité d’éthique Hospitalo-facultaire de Saint-Luc”. Written informed consent was obtained from all participants before inclusion in the study. The trial was registered at ClinicalTrials.gov under identification number NCT03852069 and the biological data related to the intervention study have been published previously [17].

### 2.2. Anthropometric Characteristics

Weight, height, waist and hip circumference, blood pressure and body composition were measured at baseline and after three months of intervention. Body composition was assessed by bio-impedance devices (BIA 101, Akern, Italy; Biocorpus, Medi Cal, Germany; Tanita BC-418 MA, Tanita, UK). Resistance measurement was used to calculate fat-free mass and total body fat. Subcutaneous and visceral fat areas were obtained by CT-scan, and Fibroscan was used to quantify liver stiffness (elasticity) and controlled attenuation parameter.

### 2.3. Dietary Anamnesis

The dietary assessment was carried out by a trained dietician at baseline using a one-week recall questionnaire to assess the dietary intake. Energy, macronutrients and amino acid intakes were evaluated using the Canadian Nutrient File and the Souci-Fachmann-Kraut Datenbank.

### 2.4. Gut Microbiota Composition

Stool samples were collected at baseline and stored at room temperature with a DNA stabilizer (Stratecbiomolecular, Berlin, Germany) for a maximum of three days, then transferred to −80 °C. Genomic DNA was extracted using a PSP^®^ spin stool DNA kit (Stratecbiomolecular). Sequencing and subsequent bioinformatics were performed as previously described [18]. For the gut microbiota analysis, raw sequences can be accessed in Sequence Read Archive database (SRA accession numbers PRJNA595949). Clr-transformed data were used to conduct statistical analysis [19]. Metagenomics predictions based on amplicon sequence variants (ASV) were generated using PICRUSt2 [20]. The mean weighted Nearest Sequenced Taxon Index (NSTI) was 0.15.

Amplicon sequencing of the microbiome was done at the University of Minnesota Genomics Center. Briefly, the V5-V6 region of the 16S rRNA gene was PCR-enriched using the primer pair V5F_Nextera (TCGTCGGCAGCGTCAGATGTGTATAAGAGACAGRGGATTAGATACCC) and V6R_Nextera (GTCTCGTGGGCTCGGAGATGTGTATAAGAGACAGCGACRRCCATGCANCACCT) in a 25 μL PCR reaction containing 5 μL of template DNA, 5 μL of 2× HotStar PCR master mix, 500 nM of final concentration of primers and 0.025 U/μL of HostStar Taq + polymerase (QIAGEN). PCR-enrichment reactions were conducted as follow, an initial denaturation step at 95 °C for 5 min followed by 25 cycles of denaturation (20 s at 98 °C), annealing (15 s at 55 °C), and elongation (1 min at 72 °C), and a final elongation step (5 min at 72 °C). Next, the PCR-enriched samples were diluted 1:100 in water for input into library tailing PCR. The PCR reaction was analogous to the one conducted for enrichment except with a KAPA HiFi Hot Start Polymerase concentration of 0.25 U/μL, while the cycling conditions used were as follows, initial denaturation at 95 °C for 5 min followed by 10 cycles of denaturation (20 s at 98 °C), annealing (15 s at 55 °C), and elongation (1 min at 72 °C), and a final elongation step (5 min at 72 °C). The primers used for tailing are the following: F-indexing primer AATGATACGGCGACCACCGAGATCTACAC[i5]TCGTCGGCAGCGTC and R-indexing primer CAAGCAGAAGACGGCATACGAGAT[i7]GTCTCGTGGGCTCGG, where [i5] and [i7] refer to the index sequence codes used by Illumina. The resulting 10 μL indexing PCR reactions were normalized using a SequalPrep normalization plate according to the manufacturer’s instructions (Life Technologies). 20 μL of each normalized sample was pooled into a trough, and a SpeedVac was used to concentrate the sample pool down to 100 μL. The pool was then cleaned using 1× AMPureXP beads and eluted in 25 μL of nuclease-free water. The final pool was quantitated by QUBIT (Life Technologies, Merelbeke, Belgium) and checked on a Bioanalyzer High-Sensitivity DNA Chip (Agilent Technologies, Machelen, Belgium) to ensure correct amplicon size. The final pool was then normalized to 2 nM, denatured with NaOH, diluted to 8 pM in Illumina’s HT1 buffer, spiked with 20% PhiX, and heat denatured at 96 °C for 2 min immediately prior to loading. A MiSeq 600 cycle v3 kit was used to sequence the pool.

Subsequent bioinformatics and biostatistics analyses were performed *in house*. Initial quality filtering of the reads was performed with the Illumina Software, yielding an average of 99,159 pass-filter clusters per sample. Quality scores were visualized with the FastQC software (http://www.bioinformatics.babraham.ac.uk/publications.html; version 0.11.9), and reads were trimmed to 220 bp (R1) and 200 bp (R2) with the FASTX-Toolkit (http://hannonlab.cshl.edu/fastx_toolkit/; version 0.0.13). Next, reads were merged with the merge-illumina-pairs application v1.4.2 (with *p* = 0.03, enforced Q30 check, perfect matching to primers which are removed by the software, and otherwise default settings including no ambiguous nucleotides allowed) [21]. For all the samples, a subset of 25,000 reads was randomly selected using Mothur v1.25.0 [22] to avoid large disparities in the number of sequences. Subsequently, the UPARSE pipeline implemented in USEARCH [23] was used to further process the sequences. Amplicon sequence variants (ASVs) were identified using UNOISE3 [24]. Taxonomic prediction was performed using the *nbc_tax* function, an implementation of the RDP Naive Bayesian Classifier algorithm [25].

### 2.5. Non-Targeted Metabolomics

Metabolomics analysis was conducted on a subset of subjects (n = 38, 23 and 15 in High and Low mood score group respectively; from the St Luc Hospital). The non-targeted metabolomics analysis pipeline has been described in detail before [26]. Frozen plasma samples were randomized. For metabolite extraction, cold acetonitrile was added in a ratio of 400 µL per 100 µL of plasma. The samples were then vortexed for 15 s, sonicated for 5 min, and centrifuged for 5 min at 4 °C and 13,000 rpm. The samples were kept in ice between the steps. The supernatants were filtered (Acrodisc 4 mm with 0.45 µm membrane) and inserted into HPLC vials for analysis. The QC sample was prepared by collecting 10 µL from each sample vial and combining the material in another vial.

The samples were analyzed by liquid chromatography–mass spectrometry, consisting of a 1290 Infinity Binary UPLC coupled with a 6540 UHD Accurate-Mass Q-TOF (Agilent Technologies). A Zorbax Eclipse XDB-C18 column (2.1 × 100 mm, 1.8 µm; Agilent Technologies) was used for the reversed-phase (RP) separation and an Aqcuity UPLC BEH amide column (Waters) for the HILIC separation. After each chromatographic run, the ionization was carried out using jet stream electrospray ionization (ESI) in the positive and negative mode, yielding four data files per sample. The collision energies for the MS/MS analysis were selected as 10, 20 and 40 V, for compatibility with spectral databases.

Peak detection and alignment was performed in MS-DIAL ver. 3.96 [27]. For the peak collection, m/z values up to 1500 and all retention times were considered. The amplitude of minimum peak height was set at 2000. The peaks were detected using the linear weighted moving average algorithm. For the alignment of the peaks across samples, the retention time tolerance was 0.05 min and the m/z tolerance was 0.015 Da. Drift correction and removal of low quality signals was done as described previously [26].

The chromatographic and mass spectrometric characteristics (retention time, exact mass, and MS/MS spectra) of the significantly differential molecular features were compared with entries in an in-house standard library and publicly available databases, such as METLIN and HMDB, as well as with published literature. The annotation of each metabolite and the level of identification was given based on the recommendations published by the Chemical Analysis Working Group (CAWG) Metabolomics Standards Initiative (MSI) [28]: 1 = identified based on a reference standard, 2 = putatively annotated based on MS/MS spectra or physicochemical properties, 3 = putatively annotated to a compound group (e.g., phosphatidylcholine) and 4 = unknown.

### 2.6. Psychological Measures

Participants were asked to answer to semi-structured interview questions regarding their background information and lifestyle, fill out self-reported questionnaires, and perform cognitive tasks on a computer before and after the intervention. The following self-reported questionnaires were used to measure actual and general mood, and emotion regulation abilities: Positive and Negative Affect Schedule (PANAS; NA and PA, negative and positive affect respectively), the Scale of Positive and Negative Experience (SPANE, NE and PE, negative and positive emotion respectively; BE, Balance emotion), and the Profile of Emotional Competences (PEC. TOT, total, INTRA, intrapersonal; INTER, interpersonal, Self Reg, emotional self-regulation) [29,30,31].

### 2.7. Statistical Analyses

R Software (version 3.5.1, MixOmics package), JMP Pro 14, and Graphpad Prism 8.0 were used for analyses. Mood score was based on the positivity score (Positive scale of the PANAS-Negative scale) as previously described [18]. Segregation was made using the median of the positivity score: individuals with a higher positivity score were assigned to the “High mood score” group while those with lower score were assigned to the “Low mood score” group and were presented as “High” and “Low” in all tables and figures.

**Figure 1:**
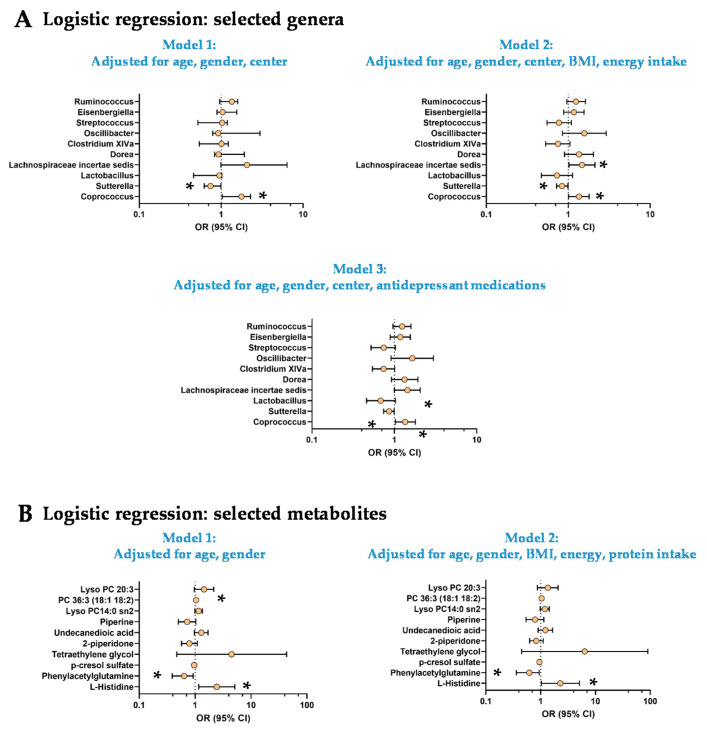
Logistic regressions for selected genera and metabolites. Logistic regression with TOP10 microbial genera (**A**) and metabolites (**B**). Odd ratio and 95% confidence intervals were represented. * significant results (*p* < 0.05). A. Model 1: Logistic regression adjusted for age, gender and center; Model 2: Logistic regression adjusted for age, gender, center, BMI, energy intake; Model 3: Logistic regression adjusted for age, gender, center and antidepressant medications. B. Model 1: Logistic regression adjusted for age and gender; Model 2: Logistic regression adjusted for age, gender, BMI, energy and protein intake. PC: Phosphatidylcholine.

Group differences were assessed using χ^2^-tests for categorical variables and parametric t-tests when applicable or Mann-Whitney-Wilcoxon tests for quantitative variables based on data distribution (Shapiro-Wilk test). Logistic regressions were used to confirm the robustness of the observations by taking into account major potential confounding factors for each type of variables. All models were adjusted for age, gender and the center (except for metabolomics data available for one center). Then, depending on the type of variables, models were adjusted for BMI, nutritional habits (energy or protein intake) or the use of antidepressant medications. The different models are described in the legend of each table. Odds ratios (OR) and confidence interval (95%) were estimated for the logistic regressions and are represented in the Figure 1.

As metagenomics and metabolomics data were characterized by a large number of variables, we conducted partial least square discriminant analysis (PLS-DA) and a more restrictive approach using sPLS-DA (mixOmics). Based on the variable importance in projection (VIP) scores of the PLS-DA we defined the top 10 variables accounting for the difference between High and Low mood groups for bacterial genera and blood metabolites.

Analyses of correlation were conducted using the Spearman method. False discovery rate (FDR) approach was used to calculate q-value by using the Benjamini, Krieger and Yekuteli method. Data are expressed as mean ± SD. *p* and q-value were considered as statistically significant when *p* < 0.05. 

## 3. Results

### 3.1. Mood Status Characterization and Related Psychological and Behavioural Profiles

The positivity score, based on the difference between the positive and the negative scale of the PANAS score, allows us to segregate obese subjects in two populations (median value: 16): one with high positivity score (24.5 ± 5.2, n = 47; “High mood score group”), and another low positivity score (7.8 ± 8.4, n = 47; “Low mood score group”) (Table 1). There were no statistically significant differences in sociodemographic characteristics or medication use in High vs. Low mood subjects except a difference in family status, namely a higher rate of non-married or attached individuals in the Low mood group (Supplementary Table S1).

### 3.2. The Low Mood Score Group Did Not Display Specific Clinical Features

Univariate analysis revealed that among anthropometric, biological and nutritional parameters, the Low mood score group did not display any significant difference compared to the High mood score group (Supplementary Table S2). Model 1 (logistic regression adjusted for age, gender and center) confirmed these results while model 2 (logistic regression adjusted for age, gender, center, BMI and energy intake) only revealed a lower protein intake in Low mood score group versus High mood score group.

### 3.3. The Low Mood Score Group Are Characterized by Specific Gut Microbiome Composition

PLS-DA was used to select bacterial genera responsible for the segregation of Low and High mood score groups (Supplementary Table S3; n = 86 individuals, Supplementary Figure S1). It allowed to select a top 10 of the genera based on their VIP score. Univariate analysis revealed that the Low mood score group displayed elevated levels of *Coprococcus* and lower levels of *Sutterella* and *Lactobacillus* (Supplementary Table S3). None of these differences remained significant after FDR corrections. We used three logistic regression models adjusted for age, gender and center (model 1) + BMI and energy intake (model 2) or antidepressant use (model 3; Figure 1A). The models 2 and 3 revealed a significantly higher abundance of *Lachnospiraceae incertae sedis* in the Low mood score group (OR: 1.47 95% CI [1.02–2.12]; OR = 1.44, 95% CI [1.00–2.06] respectively; Figure 1A). There were no more statistical differences in the abundance of *Lactobacillus* after adjustments in the three models. Despite elevated VIP scores in the PLS-DA and sPLS-DA analysis and trends (*p* < 0.10) in Mann-Whitney tests or logistic models, the other genera (*Dorea, Clostridium XIVa, Oscillibacter, Streptococcus, Eisenbergiella* and *Ruminococcus*) were not different between both groups (Figure 1A and Supplementary Table S3).

We used the same model in the subpopulation of individuals with available metabolomics data (Supplementary Table S4, n = 38) to test whether the observations made in the whole cohort can be replicated. This was the case for *Coprococcus* and *Lactobacillus* while for *Sutterella*, the difference did not reach significance (*p* = 0.09, Mann-Whitney test; Supplementary Table S4). A significantly lower levels of *Clostridium XIVa* was observed in the Low mood score group in all the models used (Supplementary Table S4), which was not the case when the whole cohort was considered (Figure 1A).

### 3.4. The Low Mood Score Group Exhibited Selective Profile of Plasma Metabolites

PLS-DA and sPLS-DA were used to select the Top 10 plasma metabolites that discriminate Low and High mood score groups (Supplementary Table S5; n = 38 individuals, Supplementary Figure S1). Among them, we found several amino acids and derivatives such as L-histidine, phenylacetylglutamine, p-cresol sulfate or 2-piperidone (δ-valerolactam, Supplementary Table S5). Several (lyso)phosphatidylcholines (PC) were also pivotal to segregate Low and High mood score groups (Supplementary Table S5). Univariate analysis revealed a higher L-histidine levels and lower levels of phenylacetylglutamine in Low mood score group versus the High mood score groups (Supplementary Table S5). None of the differences reached the statistical significance after FDR correction (Supplementary Table S5). The logistic regression adjusted for age and gender (Model 1) and age, gender, BMI, energy and protein intake (Model 2) confirmed the robustness of the changes observed for L-histidine and phenylacetylglutamine (Figure 1B). After adjustment for age, gender, BMI, energy and protein intake (model 2) PC 36:3 (18:1–18:2) was significantly higher in the Low mood score group (Figure 1B).

### 3.5. Origins of the Differences in L-Histidine and Phenylacetylglutamine Levels

Recent work suggested that nutrition, gut microbiota composition and function as well as clinical features were the strongest predictors of the circulating metabolome [16]. Thus, we wanted to explore the importance of each component in the levels of L-histidine and phenylacetylglutamine.

**Figure 2:**
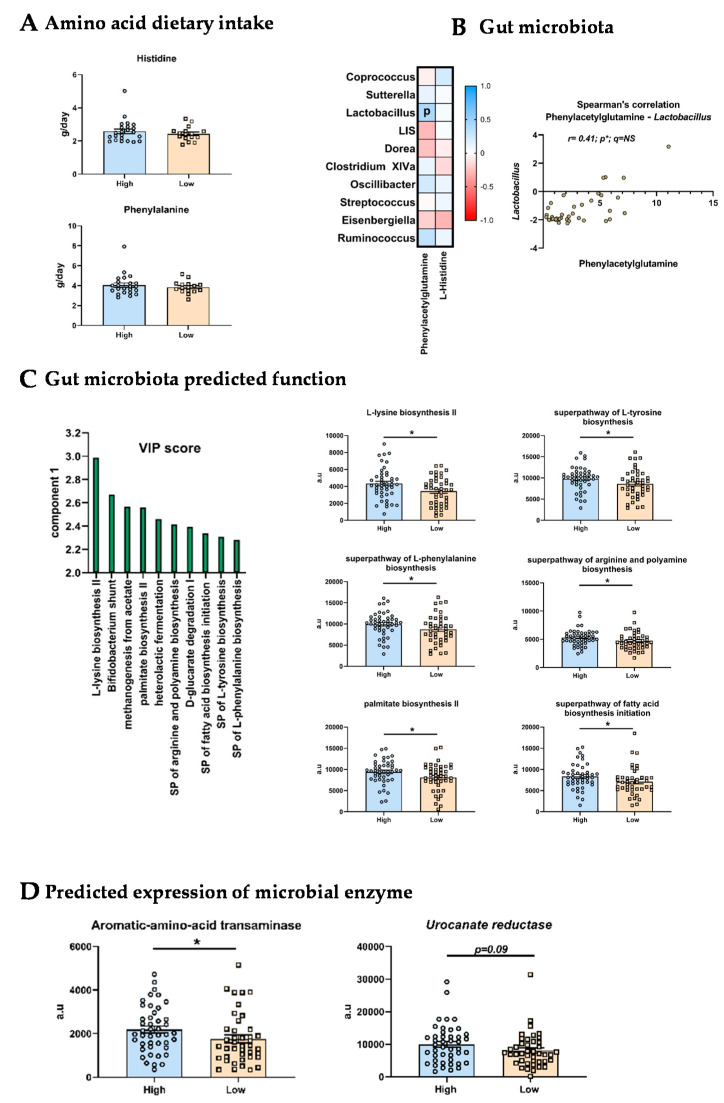
Origins of the altered serum metabolite profile. (**A**) Dietary intake of amino acids in High and Low mood score groups. (**B**) Spearman’s correlation matrix between metabolites and genera of interest. Significant (*p* < 0.05) correlation were highlighted with “p”. (**C**) PICRUSt2 analysis. Left part: graphical representation of the TOP10 MetaCyc pathways which segregate High and Low mood score groups (based on PLS-DA VIP scores). Right part: graphical representation of the six significantly different MetaCyc pathways between High and Low mood score groups. a.u: arbitrary unit (**D**) PICRUSt2 analysis. Graphical representation of the predicted expression of the aromatic amino-acid transaminase and the urocanate reductase. * *p* < 0.05.

Dietary anamnesis revealed that there were no differences in the intake of histidine and phenylalanine from dietary proteins between low mood and high mood groups (Figure 2A).

Then, we performed correlation between the clr value of the top 10 genera and the levels of the two most discriminant metabolites between both groups: L-histidine and phenylacetylglutamine (Figure 2B). We observed only one significant correlation between the phenylacetylglutamine levels and the *Lactobacillus* abundance (*r* = 0.41; *p* = 0.01), which was no more significant after FDR correction. Histidine levels were not significantly associated with any of the microbial genera (Figure 2B).

Besides the gut microbiota composition, we assessed predicted function from microbiome analysis by using PICRUSt2. Regarding MetaCyc pathways, a PLS-DA analysis allowed us to select the TOP10 pathways pivotal for the segregation of High and Low mood score groups (Figure 2C). Among these pathways, univariate analysis revealed that six were significantly different between both groups. Interestingly, we observed that the biosynthesis pathways of several amino acids including the L-tyrosine and L-phenylalanine were predicted to be significantly less functional in the Low mood score group. Then, we explored individually the predicted expression of the enzymes involved in the metabolism of histidine and phenylalanine. The aromatic amino acid transaminase, which allows initiating the transformation of phenylalanine into phenylacetate (further transformed by the liver into phenylacetylglutamine), was predicted to be significantly less expressed in the gut microbiota of the Low mood score group (Figure 2D). Regarding histidine metabolism, we found no difference in any of the predicted expression of the enzymes but only non-significant decrease (*p* = 0.09) for the urocanate reductase, an enzyme involved in the production of imidazole propionate from histidine, in the low mood score group (Figure 2D).

The accumulation of some metabolites in the blood -especially phenylacetylglutamine- can result from disturbances of its clearance by kidneys observed for example in chronic kidney disease [32]. Based on our metabolite profiling, we confirmed that kidney function did not appear to be affected since urea and creatinine levels were comparable between both groups (Supplementary Figure S2).

### 3.6. Relationship between the Selected Microbial Genera

**Figure 3:**
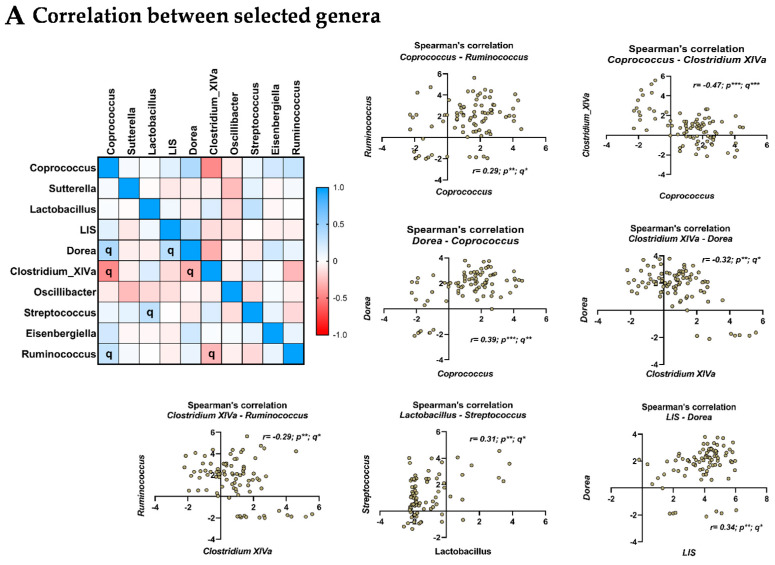

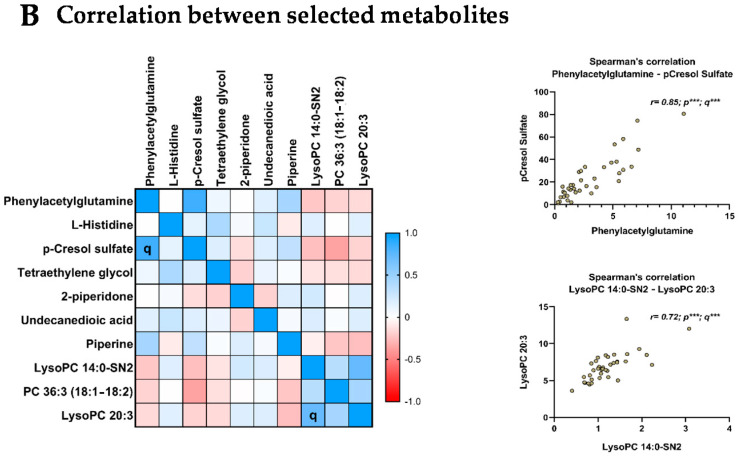
Relationship between the selected microbial genera and metabolites. (**A**) Spearman’s correlation matrix between genera of interest (TOP10). Significant (q < 0.05) correlation were highlighted with “q”. Individual significant correlation were represented in the right part. (**B**) Spearman’s correlation matrix between metabolites of interest (TOP10). Significant (q < 0.05) correlation were highlighted with “q”. Individual significant correlation were represented in the right part. * *p* or q < 0.05; ** *p* or q < 0.01; *** *p* or q < 0.001.

As it is now well recognized that some bacteria cooperate to grow notably through cross-feeding we assessed if the abundance of some genera was associated with others. To do so we performed a Spearman’s correlation matrix (Figure 3A). We observed that several genera were correlated with each other (q-value < 0.05). *Coprococcus* was positively associated with *Ruminococcus* and *Dorea* while it was negatively correlated to *Clostridium XIVa* (Figure 3A). *Clostridium XIVa* was negatively associated with *Ruminococcus* and *Dorea* (Figure 3A). Positive associations were found between the abundances of *lactobacillus* and *streptococcus* as well as between *lachnospiraceae incertae sedis* and *dorea* (Figure 3A).

### 3.7. Relationship between the Selected Metabolites

Regarding circulating metabolites two correlations were found. We observed a significant (q < 0.05) positive correlation between the two amino-acids derivates phenylacetylglutamine and p-Cresol sulfate (Figure 3B). We also observed that the lyso PC 20:3 was positively associated with lyso PC 14:0 sn-2.

## 4. Discussion

This study reveals that mood disturbances in obese subjects are associated with specific alterations of gut bacteria and plasmatic metabolites. Despite the fact that the circulating metabolite levels and microbiome signatures did not reveal drastic changes between low and high mood obese patients, the statistical analysis revealed very specific features associated with mood in the cohort. *Sutterella* and *Lactobacillus* were lower in obese individuals with mood disturbances while *Coprococcus* was higher. Regarding plasma metabolite profile, the multivariate analysis revealed that several protein-derived metabolites (histidine, phenylacetylglutamine, p-cresol sulfate, 2-piperidone) and lipid metabolites (Lyso PC14:0 sn2 and lyso PC 20:3 and PC 36:3) distinguished the two groups of patients. After adjustments for several confounding factors, only increased levels of L-histidine and decreased levels of phenylacetylglutamine characterized the obese subjects with mood disturbances, who did not present other anthropometric or biological characteristics.

Phenylacetylglutamine has been highlighted as a biomarker of healthy aging, and being associated with a shift in microbiome composition [33]. This last study confirmed previous data showing elevated levels of this metabolite in centenarians [34]. This metabolite has also been associated with an increased α-diversity and abundance of putatively “beneficial” bacteria (*Akkermansia muciniphila*, genus from *Christensenellaceae*) despite its potential detrimental effects on cardiovascular health [35,36]. Little is known regarding phenylacetylglutamine relationship with psychiatric symptoms. Two studies found elevated levels of phenylacetylglutamine in patients suffering from depression after stroke or anorexia nervosa [37,38].

The other metabolite that was pivotal in segregating obese individuals with or without mood disturbances was histidine. This essential amino acid is crucial for several physiological processes, including immunomodulation, the scavenging of reactive oxygen species or proton buffering [39]. Histidine is the precursor of several bioactive compounds including histamine [39,40]. Regarding brain function, a preclinical study suggested that histidine deficiency can lead to brain histamine depletion which is associated with anxiety [41]. Moreover, through the immunomodulatory effects of histamine, the histidine supplementation can exert neuroprotective effect in the context of epilepsy [42]. In humans, histidine supplementation was associated with improvements in mental fatigue and cognition [43]. On the contrary, high doses of histidine supplementation can lead to detrimental effect in healthy subjects such as confusion or depression [44]. Overall, there is no clear evidence of a relationship between mood and physiological blood levels of histidine, especially in the context of obesity. Unlike for phenylacetylglutamine, our data did not allow to speculate on the origins of the difference seen in histidine circulating levels. Further studies are needed to better understand why there is an increased level of histidine in obese individuals with mood disturbances and whether and how it can affect their behaviour.

The bacterial genera associated with mood in our study have already been associated with metabolic alterations and behavioural disorders in other studies. The higher level of *Coprococcus* associated with mood disorders is of particular interest. We and other have already shown that *Coprococcus* can be an interesting genus to study when it comes to emotional regulation [18,45]. Our previous study revealed that elevated levels of *Coprococcus* in obese individuals predict a better response toward inulin supplementation regarding their mood scores [18]. Bacteria from this genus are well-recognized butyrate producers, which is interesting since this SCFA has been shown to exert neuroprotective and antidepressant effects in preclinical models [46]. Valles-Colomer and colleagues postulated that it could be involved in neurotransmitters metabolism (3,4-dihydroxyphenylacetic acid-DOPAC-synthesis) based on correlative analysis [45].

*Sutterella*, for which levels are lower in obese individuals presenting mood alterations, is composed of three anaerobic species (*S. parvirubra, S. stercoricanis, and S. wadsworthensis*). Its occurrence has already been shown in a recent systematic review to decrease in depression [47]. A recent report revealed that this genus also decreased in diabetic patients while its increase was associated with improvement of glycemia after Roux-en-Y bariatric surgery [48]. *Sutterella* has also been highlighted in the field of inflammatory bowel diseases (IBD) -with conflicting results- [49]. This genus remains understudied and little is known regarding the mechanisms through which *Sutterella* influences the host. *Sutterella* exerts some immunomodulatory effects as it has been negatively associated with cytokines such as IL-12 or IL-13 in patients suffering IBD and is able to degrade IgA [49,50,51]. *Sutterella* is a bacteria that does not use carbohydrates as energy source but which is able to degrade protein substrates [52]. This characteristic is particularly interesting in our context since protein-derived metabolites were the more important to segregate obese subjects with mood disturbances. Of interest, protein degraders seem to compete and to be mutually exclusive in culture based on protein as a main substrate [52].

Two others genera display weaker associations with mood status in our study: *Lactobacillus* and *Lachnospiraceae incertae sedis*, which abundances were lower and higher in the obese patients presenting mood alterations in our cohort respectively. The former can influence brain function and behaviour [5,53,54]. Some *Lactobacillus* species (*L. fermentum* and *L. acidophilus*) have been associated with blood metabolite composition in a large study conducted in general population by Visconti and colleagues [55]. Interestingly, it appears that several *Lactobacillus* species were positively associated with phenylpyruvate and phenylacetylglutamine [55]. The metabolic filiation is the following: phenylalanine is metabolized by bacteria into phenylacetate, which in its turn, is transformed in the host, mainly by the liver, into phenylacetylglutamine. In accordance, a pilot study revealed that *Lactobacillus casei shirota* supplementation in football players increased the urinary phenylacetylglutamine [56]. We observed the same positive association between *Lactobacillus* and plasma phenylacetylglutamine, both variables being positively associated with mood in our study. *Lachnospiraceae incertae sedis* has been shown to be higher in people suffering depression which is in line with our findings (higher in Low mood score group) [47,57]. We observed that among the genera involved in the segregation of our two groups of obese individuals, several were robustly correlated. For example, the genus *Coprococcus* was co-abundant with *Ruminococcus* and *Dorea* while it was negatively associated with *Clostridium XIVa*. The concept of guild in gut microbiota, which refer to a group of constantly co-abundant bacteria that are likely to be functionally related is particularly interesting to explore when it comes to study function of the gut microbiota (i.e., metabolites production) [58,59]. In future studies, with sequencing method allowing this kind guild-based analysis at the species level, it could be interesting to look for more robust associations with circulating metabolite profile and psychological symptoms of obese individuals.

Our results suggest that the differences of the phenylacetylglutamine levels can be due to changes in the gut microbiota composition (*Lactobacillus* levels) and function (predicted bacterial amino acid production and metabolism) rather than from differences in dietary intake or renal excretion capacity. It is in line with the fact that phenylacetylglutamine levels are highly associated with changes in gut microbiota composition [55]. Our observations that the microbial pathways of aromatic amino acids biosynthesis are lower in the Low mood score group is intriguing. The gut microbiota can synthesize all amino acids that are then used by the host. However, the relative contribution of this phenomenon compared to dietary intake as well as its impact on health remain unknown [60]. Aromatic amino acids are crucial for brain function as precursors of neurotransmitters and others neuroactive compounds and have been recently associated with cognition in obese individuals [14]. Thus, it appears of interest to study in more details whether changes in gut microbiota composition and function can affect amino acids metabolism (both production and catabolism), especially in the context of psychiatric disorders.

One limitation of our study is the absence of longitudinal follow-up, and hence our study does not allow any assumption on causality. Further studies are needed to understand whether specific microbes (*Coprococcus*, *Sutterella* or *Lactobacillus)* or amino-acids metabolites regulate emotion in obese individuals, namely by evaluating the proof of concept of the relevance of the selected bacteria and metabolites in other larger cohorts of obese patients, but also in non obese individuals. Indeed, an additional limitation of this study is the lack of lean individuals with high and low mood scores. Inclusion of those individuals would allow this study to determine if these metabolic differences are unique to obese individuals or to mood disorders in general. One criticism could be that the PANAS test, which is coherent to assess mood, is not a validated test of depression and anxiety. Nevertheless, the PANAS test has been shown to be associated with another test of depression [61]. The reliability of our classifications is reinforced by the marked differences observed with other tests assessing mood (SPANE scale) and emotional competence (PEC).

## 5. Conclusions

In summary, we discovered in this study that emotional disturbances in obese individuals are characterized by specific changes in gut microbiota composition (lower levels of *Sutterella* and *Lactobacillus* and higher levels of *Coprococcus*) and function (amino acid metabolism) which are translated into modifications of the level of some amino acid-derived metabolites (L-histidine and phenylacetylglutamine). Our data, if confirmed further in larger cohorts, would allow to propose new targets to tackle mood disturbances seen in obese individuals.

## Figures and Tables

**Table 1:** Psychological parameters in obese subjects with High and Low mood scores 1.

	High	Low	*p*	Model 1		Model 2	
	Mean ± SD	Mean ± SD		OR	*p*	OR	*p*
PANAS PA	36.4 ± 4.90	27.2 ± 6.36	<0.0001	0.75	<0.0001	0.75	<0.0001
PANAS NA	11.9 ± 3.26	19.5 ± 8.06	<0.0001	1.36	<0.0001	1.42	<0.0001
PANAS PA-NA	24.5 ± 5.17	7.77 ± 8.36	<0.0001	1.92 × 10^−13^	<0.0001	1.06 × 10^−6^	<0.0001
PEC TOT	3.42 ± 0.44	3.18 ± 0.47	0.012	0.26	0.006	0.26	0.016
PEC INTRA	3.37 ± 0.50	3.11 ± 0.55	0.027	0.33	0.016	0.36	0.034
PEC INTER	3.40 ± 0.49	3.20 ± 0.51	0.041	0.36	0.032	0.34	0.029
PEC Reg Self	3.26 ± 0.80	2.85 ± 0.86	0.013	0.54	0.025	0.58	0.048
SPANE.PE	20.5 ± 2.62	17.2 ± 8.77	<0.0001	0.86	0.024	0.89	0.078
SPANE.NE	10.9 ± 3.07	13.7 ± 4.03	0.0003	1.25	0.001	1.24	0.003
SPANE.BE	9.65 ± 5.09	3.53 ± 10.6	<0.0001	0.89	0.003	0.90	0.008

Unidimensional analysis revealed that Low mood group displayed lower scores in all tests related to emotion (PANAS, PEC, SPANE) (Table 1). PANAS, Positive and Negative Affect Schedule; PEC, Profile of Emotional Competences; SPANE, Scale of Positive and Negative Experience; PA, positive affect; NA, negative affect; PE, positive emotion; NE, negative emotion; BE; Balanced emotion; INTRA, intra-personal; INTER, inter-personal; REG SELF, self-regulation TOT, total.

## Data Availability

The datasets used and/or analysed during the current study are available from the corresponding author on reasonable request. The accession number for the raw data generated with the 16S rRNA gene sequencing reported in this paper is BioProject PRJNA595949 (SRA) and are available here https://www.ncbi.nlm.nih.gov/bioproject/PRJNA595949/ (access on 16 December 2019).

## Supplementary Materials

The following are available online at https://www.mdpi.com/article/10.3390/nu14010147/s1, Supplementary Methods; Supplementary Figure S1: Flow chart of the study presenting the number of patients included in each analysis; Supplementary Figure S2: Relative levels of urea and creatinine in the blood. Supplementary Table S1: Baseline characteristics of the participants; Supplementary Table S2: Biological and nutritional parameters in obese subjects with High and Low mood scores; Supplementary Table S3: TOP 10 Microbial genus discriminating obese subjects with High and Low mood scores; Supplementary Table S4: TOP 10 Microbial genus discriminating obese subjects with High and Low mood scores in the subpopulation with untargeted metabolomics; Supplementary Table S5: TOP 10 Circulating metabolites discriminating obese subjects with High and Low mood scores.
