# Supplementary material for: Microbiota and Metabolite Profiling as Markers of Mood Disorders: A Cross-Sectional Study in Obese Patients
Source: Nutrients. 2021 Dec 29;14(1):147. doi: 10.3390/nu14010147 (PMC8746987; doi:10.3390/nu14010147)
Supplement: Supplementary file 1 [file nutrients-14-00147-s001.zip › Supplementary Figures.pdf]

Flow Diagram

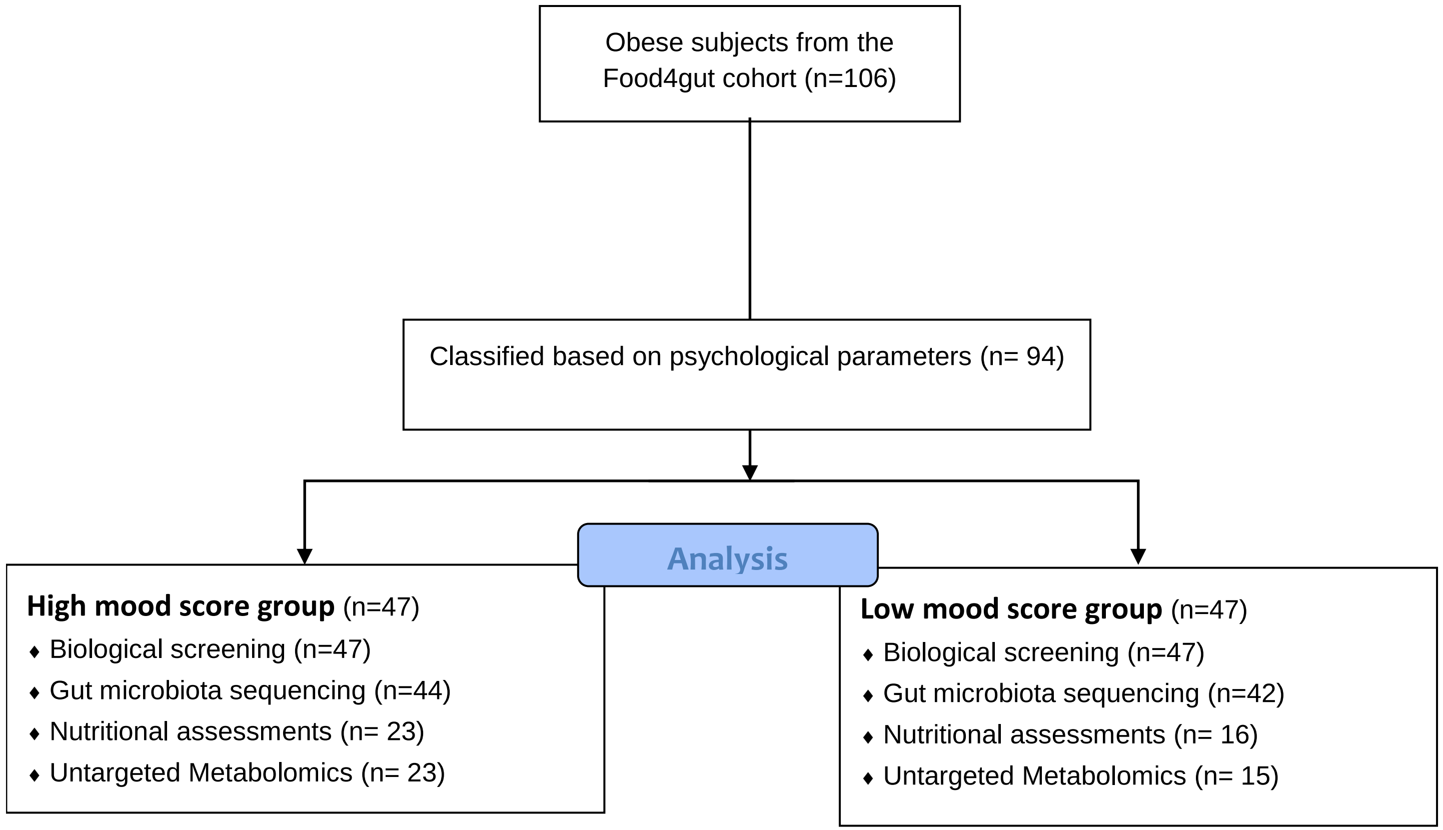

Supplementary Figure S1: Flow chart of the study presenting the number of patients included in each analysis.

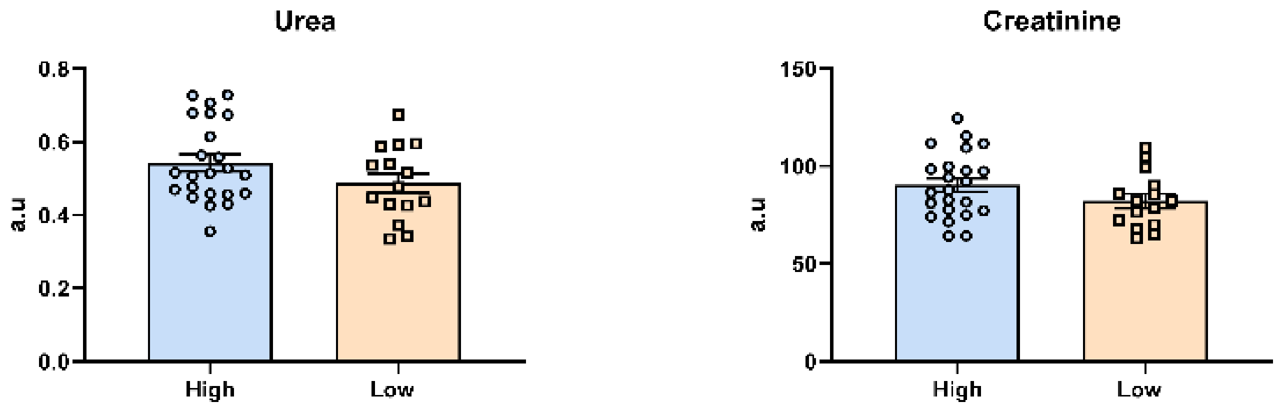

Supplementary Figure S2: Relative levels of urea and creatinine in the blood.
Data represented are mean ± SEM. Unpaired t-test or Mann-Whitney tests were used.
