# Supplementary material for: Microbiota and Metabolite Profiling as Markers of Mood Disorders: A Cross-Sectional Study in Obese Patients
Source: Nutrients. 2021 Dec 29;14(1):147. doi: 10.3390/nu14010147 (PMC8746987; doi:10.3390/nu14010147)
Supplement: Supplementary file 1 [file nutrients-14-00147-s001.zip › Supplementary Methods.pdf]

Supplementary Methods

Participants

The inclusion criteria were: BMI > 30 kg/m^2, age 18 to 65 years, Caucasian ethnicity, presence of at least one metabolic obesity-related disorder (prediabetes/diabetes, dyslipidemia, hypertension, fatty liver disease). The exclusion criteria were: use of antibiotics, pro/prebiotics, dietary fibers or any molecule that modifies intestinal transit time within 6 weeks before starting the study, pregnancy in progress or planned within 6 months, heavy psychiatric problems and/or use of antipsychotics, recent (<6 weeks) or current particular diets (e.g., vegetarian, vegan, high-protein, high-fiber diet), excessive alcohol consumption (more than 3 drinks/day), type 1 diabetes, general dislike for vegetables. Recruitment took place from January 2016 to May 2018.

Blood parameters

Fasting glycaemia, HbA1c, liver enzymes, and lipids were measured in the hospital laboratory. The remaining plasma was centrifuged at 2000 g for 10 min at 4°C and frozen at -80°C. Insulin levels were measured by ELISA (Mercodia, Uppsala, Sweden). Dipeptidyl-peptidase IV (DPP-IV) activity was assessed as previously described [1]. C-reactive protein (CRP) levels were measured using the quantikine ELISA (R&D Systems, Minneapolis, USA).

Psychological assessments

1. Interview questionnaires

To collect personal data and background information regarding circumstances of obesity, family situation and lifestyle we used a semi-structured interview.

2. Questionnaires

To measure mood, stress, self-regulation and emotional competences, and eating problems, participants filled out the following questionnaires.

1. Profile of Emotional Competences consists in 50 items to measure intra-personal and inter-personal emotion intelligence separately. Each item is rated on a 5 points scale-rating from 1 (never) to 5 (very often).
2. Positive and Negative Affect Schedule (PANAS) consists of two 10-item scales to measure both positive and negative affect. Each item is rated on a 5-point scale of 1 (not at all) to 5 (very much). The negative affect sub-scale of the PANAS strongly correlated with scores from measures related to depression and distress
3. Scale of Positive and Negative Experience (SPANE) consists of 12 items assessing separately the positive feelings (6 items), negative feelings (6 items), and the two can be combined to create a balance score. Each item is scored on a scale ranging from 1 (very rarely or never) to 5, (very often or always). The scale converges well with measures of emotions and affective well-being.

Reference :

1. Olivares M, Neyrinck AM, Pötgens SA, Beaumont M, Salazar N, Cani PD, *et al.* (2018): The DPP-4 inhibitor vildagliptin impacts the gut microbiota and prevents disruption of intestinal homeostasis induced by a Western diet in mice. *Diabetologia* 61: 1838–1848.
