# Supplementary material for: Microbiota and Metabolite Profiling as Markers of Mood Disorders: A Cross-Sectional Study in Obese Patients
Source: Nutrients. 2021 Dec 29;14(1):147. doi: 10.3390/nu14010147 (PMC8746987; doi:10.3390/nu14010147)
Supplement: Supplementary file 1 [file nutrients-14-00147-s001.zip › Supplementary Tables.pdf]

Supplementary Table S1 Baseline characteristics of the participants[1]

	High	Low	P-value
Participants (F/M)	47 (26/21)	72 (31/16)	0.29
Age, years	51.4 ± 11.1	50.5 ± 10.3	0.51
Educational level, years	14.9 ± 3.75	15.0 ± 3.31	0.28
Family status (M-A vs Non M-A)	47(38/9)	46(26/20)	**0.01**
Alcohol intake, unit / week	3.38 ± 4.94	3.04 ± 4.97	0.79
Tobacco (Yes/No)	47(5/42)	46(7/39)	0.51
Anti-depressant (Yes/No)	47(9/38)	47(11/36)	0.61
Anxiolytic (Yes/No)	47(3/44)	47(7/40)	0.18

[1]Values are means ± SD. Baseline data were analyzed by unpaired t-test or Mann Whitney for continuous variables and Chi-square tests for categorical variables. M-A: Married or Attached. F/M : female/male ; PS: Positivity score.

Supplementary Table S2 Biological and nutritional parameters in obese subjects with High and Low mood scores [1]

	High	Low		Model 1		Model 2	
	**Mean ±SD**	**Mean ±SD**	*p*	*OR*	*p*	*OR*	*p*
BMI, kg/m²	36.7 ± 5.68	35.4 ± 3.90	0.299	0.93	0.160	-	-
Fat mass, kg	38.8 ± 7.97	39.8 ± 7.66	0.537	0.98	0.471	1.01	0.765
Waist, cm	115 ± 14.9	111 ± 12.4	0.155	0.99	0.520	1.04	0.359
Hip, cm	121 ± 14.3	118 ± 11.0	0.336	0.98	0.317	0.99	0.756
Waist/hip ratio	0.96 ± 0.09	0.95 ± 0.09	0.558	5.61	0.644	4.38	0.699
Visceral fat, cm²	256 ± 109	210 ± 87.9	0.088	0.99	0.123	0.99	0.177
Subc. fat, cm²	354 ± 139	363 ± 122	0.781	1.00	0.594	1.00	0.872
Diastolic BP, mm Hg	84.7 ± 11.1	85.6 ± 11.4	0.748	1.01	0.675	1.01	0.604
Systolic BP, mm Hg	136 ± 13.6	138 ± 17.3	0.617	1.01	0.508	1.01	0.475
Cholesterol	185 ± 45.1	199 ± 49.5	0.142	1.01	0.263	1.01	0.323
HDL	45.3 ± 11.2	48.3 ± 10.6	0.199	1.01	0.473	1.01	0.626
LDL	108 ± 43.1	123 ± 42.8	0.124	1.01	0.224	1.01	0.234
Triglycerides	165 ± 94.9	160 ± 77.2	0.959	1.00	0.438	1.00	0.387
Elasticity	6.59 ± 2.71	7.36 ± 5.15	0.644	1.05	0.501	1.11	0.282
AST, U/l	26.3 ± 10.3	27.3 ± 16.7	0.660	1.01	0.658	1.00	0.965
ALT, U/l	35.1 ± 22.3	39.7 ± 27.7	0.485	1.01	0.207	1.01	0.336
γGT, U/l	44.3 ± 36.4	45.5 ± 42.6	0.808	1.00	0.872	1.00	0.947
APO-A1	1.42 ± 0.24	1.49 ± 0.23	0.244	2.38	0.408	1.88	0.558
DPP-IV, mUI/ml	17.9 ± 6.72	18.8 ± 6.58	0.222	1.02	0.536	1.02	0.580
CRP, mg/l	3.87 ± 4.73	3.95 ± 3.84	0.633	1.00	0.627	1.00	0.953
Glycemia, mg/dl	113 ± 35.8	115 ± 45.5	0.795	1.00	0.810	1.00	0.666
Insulin, mU/L	16.6 ± 10.0	15.8 ± 11.5	0.442	1.00	0.831	1.00	0.997
C-peptide, mU/L	1087 ± 477	1058 ± 497	0.804	1.00	0.865	1.00	0.429
HOMA (IR)	5.02 ± 4.45	4.62 ± 4.73	0.776	0.98	0.719	0.99	0.828
Energy, kcal/d	2058 ± 477	2064 ± 624	0.761	1.00	0.872	1.00	-
Protein, g/d	91.5 ± 20.5	84.7 ± 20.1	0.093	0.98	0.151	0.97	**0.043**
Lipid, g/d	85.1 ± 33.2	85.9 ± 41.0	0.741	1.00	0.988	0.99	0.601
Carbohydrates, g/d	211 ± 50.2	222 ± 67.2	0.372	1.00	0.277	1.01	0.109
Dietary fiber, g/d	23.7 ± 8.34	21.4 ± 9.09	0.061	0.97	0.265	0.97	0.258

[1] Unpaired t-tests or Mann-Whitney tests were used to compare to two groups; Model 1: Logistic regression adjusted for age, gender and center; Model 2: Logistic regression adjusted for age, gender, center, BMI and energy intake. BMI, body mass index; HDL, LDL, high and low density lipoprotein; ALT, alanine aminotransferase; AST, aspartate aminotransferase; γGT, γ-glutamyl transferase; APO-A1: Apolipoprotein-A1; W/H ratio: waist to hip ratio; Subc, subcutaneous: BP: blood pressure; CRP, C-reactive protein; DBP, diastolic blood pressure; DPP-IV, dipeptidyl-peptidase IV; HOMA-IR, homeostasis model assessment of insulin resistance; LDL, Low-density lipoprotein; SBP, systolic blood pressure.

Supplementary Table S3 TOP 10 Microbial genus discriminating obese subjects with High and Low mood scores [1]

	High	Low	*p*	*q*	PLS-DA VIP score	sPLS-DA VIP score
	Mean ±SD	Mean ±SD				
Coprococcus	0.82 ± 1.75	1.67 ± 1.59	**0.011**	NS	2,36	3.68
Sutterella	2.45 ± 3.03	0.99 ± 2.97	**0.020**	NS	2,27	3.43
Lactobacillus	-0.94 ± 1.51	-1.52 ± 1.10	**0.033**	NS	2,05	2.83
Lachnospiraceae incertae sedis	3.61 ± 1.55	4.19 ± 1.07	0.091	NS	2,03	2.79
Dorea	1.53 ± 1.39	2.05 ± 1.24	0.070	NS	1,84	2.27
Clostridium XIVa	1.12 ± 1.79	0.53 ± 1.30	0.154	NS	1,78	2.10
Oscillibacter	3.65 ± 0.85	3.95 ± 0.74	0.207	NS	1,77	2.07
Streptococcus	1.34 ± 1.48	0.88 ± 1.28	0.172	NS	1,56	1.51
Eisenbergiella	1.71 ± 1.63	2.22 ± 1.64	0.216	NS	1,49	1.32
Ruminococcus	1.38 ± 1.95	1.94 ± 1.78	0.299	NS	1,42	1.14

[1]Values are clr (calculated from counts – asv methods). The genus presented here were the 10 with the higher contribution for the segregation between subjects with High and Low mood scores (based on VIP scores, PLS-DA). Mann-Whitney tests were used to compare the two groups and q-value were calculated using the two-stage step-up method of Benjamini, Krieger and Yekutieli (False discovery ratio – FDR). VIP scores presented were obtained using classical or sparse partial least square discriminant analysis (PLS-DA and sPLS-DA).

Supplementary Table S4 TOP 10 Microbial genus discriminating obese subjects with High and Low mood scores in the subpopulation with untargeted metabolomics [1]

	High	Low	*p*	Model 1		Model 2		Model 3	
	**Mean ±SD**	**Mean ±SD**		***OR***	***p***	***OR***	***p***	***OR***	***p***
Coprococcus	0.46 ± 1.66	2.03 ± 1.09	**0.002**	2.31	**0.012**	2.25	**0.025**	2.29	0.014
Sutterella	2.83 ± 3.17	1.57 ± 2.86	0.094	0.87	0.232	0.83	0.137	0.85	0.174
Lactobacillus	-0.95 ± 1.29	-1.65 ± 0.81	**0.038**	0.44	0.093	0.42	0.082	0.44	0.092
Lachnospiraceae incertae sedis	3.55 ± 1.73	3.91 ± 1.26	0.679	1.14	0.607	1.24	0.420	1.21	0.457
Dorea	1.27 ± 1.56	2.14 ± 0.88	0.129	1.87	0.091	1.73	0.217	1.85	0.115
Clostridium XIVa	1.45 ± 1.94	-0.17 ± 1.04	**0.007**	0.46	**0.019**	0.39	**0.025**	0.45	**0.021**
Oscillibacter	3.54 ± 0.96	3.87 ± 0.48	0.286	2.00	0.165	1.77	0.303	1.89	0.224
Streptococcus	1.08 ± 1.42	0.64 ± 1.29	0.425	0.76	0.302	0.80	0.420	0.77	0.348
Eisenbergiella	1.41 ± 1.56	2.08 ± 2.11	0.359	1.22	0.304	1.16	0.453	1.33	0.199
Ruminococcus	1.75 ± 1.93	2.57 ± 1.32	0.314	1.34	0.193	1.25	0.413	1.35	0.212

[1]Values are clr (calculated from counts – asv methods). The genus presented here were the 10 with the higher contribution for the segregation between subjects with High and Low mood scores (based on VIP scores, PLS-DA). Mann-Whitney tests were used to compare the two groups. Model 1: Logistic regression adjusted for age, gender and center; Model 2: Logistic regression adjusted for age, gender, center, BMI, energy intake; Model 3: Logistic regression adjusted for age, gender, center and antidepressant medications. n=38.

Supplementary Table S5 TOP 10 Circulating metabolites discriminating obese subjects with High and Low mood scores [1]

	High	Low	*p*	*q*	PLS-DA VIP score	sPLS-DA VIP score
	**Mean ±SD**	**Mean ±SD**				
L-Histidine	0.81 ± 0.11	0.92 ± 0.16	**0.012**	NS	2.55	10.52
Phenylacetylglutamine	3.81 ± 2.71	1.87 ± 1.58	**0.018**	NS	2.47	9.31
p-cresol sulfate	28.3 ± 21.8	15.8 ± 11.6	0.068	NS	2.06	2.89
Tetraethylene glycol	0.12 ± 0.03	0.23 ± 0.25	0.051	NS	2.05	2.67
2-piperidone	5.92 ± 7.23	2.12 ± 1.46	0.053	NS	2.03	2.39
Undecanedioic acid	2.24 ± 0.30	2.41 ± 0.20	0.055	NS	2.02	2.28
Piperine	4.36 ± 3.59	2.44 ± 1.29	0.145	NS	2.02	2.22
Lyso PC14:0 sn2	1.09 ± 0.27	1.41 ± 0.70	0.055	NS	2.02	2.19
PC 36:3 (18:1 18:2)	119 ± 22.1	135 ± 29.3	0.059	NS	1.98	1.68
Lyso PC20:3	119 ± 22.1	135 ± 29.3	0.059	NS	1.98	1.60

[1] Unit is Signal counts x 10^5. The 10 metabolites presented here were the ones with the 10 higher VIP score in the PLS-DA analysis (comparison between subjects with High and Low mood scores).* PC: Phosphatidylcholine. Unpaired t-tests or Mann-Whitney tests were used to compare the two groups and q-value were calculated using the two-stage step-up method of Benjamini, Krieger and Yekutieli (False discovery ratio – FDR). VIP scores presented were obtained using classical or sparse partial least square discriminant analysis (PLS-DA and sPLS-DA).
